# The Flame Retardant and Mechanical Properties of the Epoxy Modified by an Efficient DOPO-Based Flame Retardant

**DOI:** 10.3390/polym16050631

**Published:** 2024-02-26

**Authors:** Pengyu Li, Jihui Wang, Changzeng Wang, Chengxin Xu, Aiqing Ni

**Affiliations:** 1 School of Materials Science and Engineering, Wuhan University of Technology, Wuhan 430070, China; 15172518737@163.com (P.L.); jhwang@whut.edu.cn (J.W.); czwang@whut.edu.cn (C.W.); xu226316@163.com (C.X.); 2 State Key Laboratory of Advanced Technology for Material Synthesis and Processing, Wuhan University of Technology, Wuhan 430070, China

**Keywords:** epoxy resin, phosphorus, flame retardancy, DOPO, high efficiency

## Abstract

Currently, the mechanical performance reduction caused by excessive phosphorus content in the halogen-free flame-retardant EP has been an obstacle to its extensive application. This study presents the effective synthesis of a novel flame-retardant BDD with great efficiency, achieving an optimum phosphorus level of merely 0.25 wt %. The structure of BDD was verified by FTIR, ^1^H NMR, ^31^P NMR and XPS spectra. To investigate the flame-retardant properties of BDD, several EPs with various phosphorus levels were synthesized. The addition of phosphorus to the EP significantly increases its LOI value from 25.8% to 33.4% at a phosphorus level of 0.25 wt%. Additionally, the resin achieves a V-0 grade in the UL 94 test. The P-HRR and THR of the modified resin measured by the cone calorimeter are also significantly reduced. At the same time, the addition of a modest quantity of BDD has a minimal impact on the mechanical properties of epoxy resin. This study shows that the removal of hydroxyl groups significantly enhances the fire resistance of phosphate-based flame retardants, thereby providing a novel approach to synthesizing efficient flame retardants.

## 1. Introduction

Epoxy resin (EP) is a particularly valuable type of high polymer that holds significant importance in both commercial and scientific fields [1,2,3]. Unfortunately, epoxy resin has poor flame retardancy and cannot self-extinguish. When it is used in daily fields such as anticorrosion coatings, adhesives, electronic and electrical devices and engineering plastics, it has a high fire risk and seriously endangers people‘s lives [2,4]. Research on imparting flame-resistant qualities to EP has gained substantial attention [5].

Historically, EP commonly employed halogenated chemicals as its flame retardants. However, the combustion products of halogenated flame retardants are corrosive and can lead to bioaccumulation, which will inevitably cause environmental problems during use [6,7,8,9]. Recently, organic phosphorus compounds showed significant effects in the modification of EP [10]. The phosphorus-containing intrinsic flame retardant is connected to the EP by chemical bonding, thereby reducing the effect on the polymer’s process performance and mechanical properties [11]. Phosphorus-containing compounds provide flame-retardant properties for polymers by reducing the emission of flammable gases and promoting the formation of a carbon layer in the matrix [12,13]. The PO· and HPO· radicals produced by the pyrolysis of phosphorus flame retardants can grab H· and ·OH radicals, thereby reducing the degree of combustion. Furthermore, the phosphate that is generated on the polymer’s surface facilitates the creation of a carbon layer, which serves to effectively insulate against oxygen and heat in the surrounding environment [14,15].

**Figure 1:**
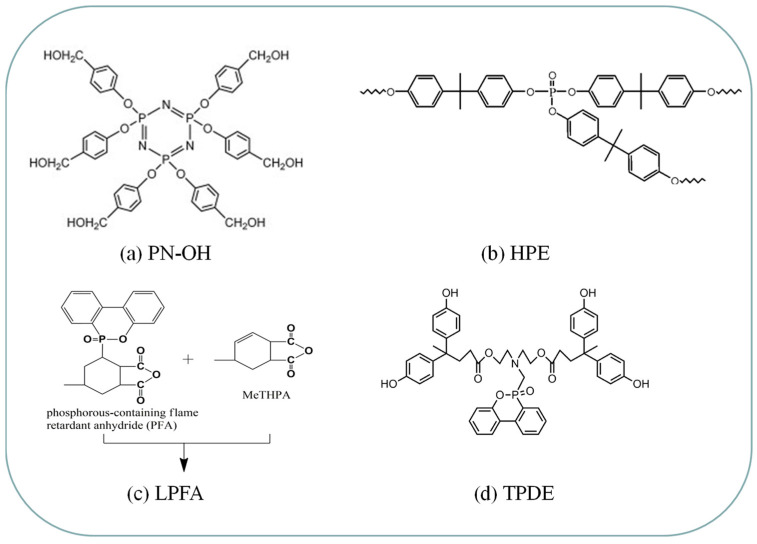
Structure diagrams of several phosphorus flame retardants.

The research on organophosphorus flame retardants mainly focuses on phosphazene, phosphonate, phosphaphenanthrene and its derivatives. The flame retardants represented by cyclotriphosphonitrile compounds have been studied and applied widely. Liu et al. [14] prepared six (4-hydroxy-phenoxy) -cyclotriphosphonitrile (PN-OH, as shown in Figure 1a) with hexachlorocyclotriphosphonitrile as raw material. A novel phosphonitrile-based epoxy resin (PN-EP) was synthesized by reacting with DGEBA. The LOI value of PN-EP is above 30%, and the UL 94 V-0 level is reached. Phosphate ester flame retardant is mainly synthesized by the reaction of phosphorus oxychloride with alcohol or phenol. Chen et al. [16] synthesized a hyperbranched polyphosphate (HPE, as shown in Figure 1b) by polycondensation of bisphenol A and phosphorus trichloride. The LOI value of the DGEBA modified by HPE is 32%, and more carbon layers are formed in the cone calorimeter test. 9,10-dihydro-9-oxa-10-phosphaphenanthrene-10-oxide (DOPO) has excellent reactivity and stability and belongs to the phosphaphenanthrene flame retardant family. Some researchers have prepared various phospho-based flame retardants based on DOPO [17,18,19]. Wu et al. [20] synthesized a new phosphorus-containing macromolecule (LPFA, as shown in Figure 1c) by combining DOPO and methyltetrahydrophthalic anhydride (MeTHPA), which reacted with EP. Results of the study on its flame retardancy showed that a UL-94 V-0 grade and an LOI value of 32.2% were obtained with a phosphorus level of 2.7 wt%. However, other important parameters, such as mechanical properties and T_g_, will be significantly affected. Chi et al. [21] employed DOPO, diethanolamine and diphenyl ether dicarboxylic acid (DPA) as starting ingredients to synthesize flame retardant (TPDE, as shown in Figure 1d) via a three-step technique. The study showed that an UL-94 V-0 rating and LOI of 42.3% were achieved with a phosphorus level of 2.32 wt%. Nevertheless, the mechanical properties of TEBA are inferior to those of DDM-cured E-51.

It is worth noting that the aforementioned thermoset resins can only obtain satisfactory flame-retardant properties with high phosphorus content, meaning that more phosphorus-containing macromolecular groups have to be incorporated. The presence of rigid structures consisting of benzene and heterophenanthrene rings will undoubtedly weaken the strength of EPs. Some researchers have also said that intrinsic flame retardants can effectively reduce the crosslinking density of polymers because the flame retardant macromolecules will combine with the epoxy group to occupy the corresponding crosslinking sites, resulting in a decrease in T_g_ [22]. As T_g_ increases, its application in the leading-edge field becomes more widespread. Consequently, the elevated phosphorus content has a negative influence on the process performance and mechanical properties of EP, thus affecting its application [23,24]. In addition, from an economic and environmental point of view, a low additive amount of flame retardant is beneficial to reducing cost and bioaccumulation [3,25]. It is necessary to prepare an excellent flame retardant for EP with a low phosphorus content in order to achieve high mechanical performance simultaneously [26,27].

In this paper, an efficient phosphorus flame retardant 6,6′-(((methylenebis(4,1-phenylene))bis(azanediyl))bis(phenylmethylene))bis(dibenzo[c,e][1,2]oxaphosphinine 6-oxide) (BDD) on the basis of DOPO was prepared by a two-step method. The structure of the BDD was determined by FTIR, ^1^H NMR, ^31^P NMR and XPS and the thermodynamics, thermal degradation, flame retardancy and mechanical properties of the BDD-modified EP were investigated. Moreover, the mechanism of phosphorus in BDD was analyzed and discussed.

## 2. Experimental

### 2.1. Materials

4,40-diamino-diphenylmethane (DDM), DOPO and benzaldehyde were provided by Macklin Biochemical Co., Ltd. (Shanghai, China). Ethanol was purchased from Wuhan Xinshentest Technology Co., Ltd. (Wuhan, China). E-51 epoxy resin (diglycidyl ether of bisphenol A, average epoxy value of 0.51 mol/100 g, molecular weight = 392, degree of oligomerization = 0.18) was supplied by Swancor (Tianjin) Wind Power Materials Co., Ltd. (Tianjin, China).

### 2.2. Synthesis of BDD

**Scheme 1:**
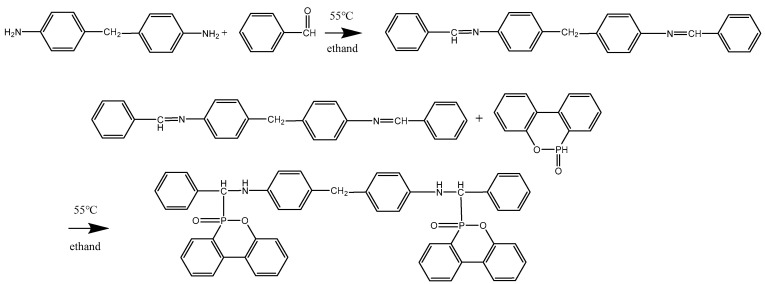
Synthesis of BDD.

DDM (15.86 g, 0.08 mol), benzaldehyde (16.98 g, 0.16 mol) and 360 mL of ethanol were put into a 1000-mL one-necked round-bottom flask and mixed at 25 °C for 30 min. The white liquid obtained was then heated and stirred at 55 °C for 2 h. The white precipitate obtained at this time is an intermediate product, Schiff base (BDM). Following a two-hour reaction period, DOPO (34.56 g, 0.16 mol) was introduced into the flask at a temperature of 55 °C and agitated for a duration of 12 h (Scheme 1). After the reaction, a white precipitate was obtained, which was then washed twice with toluene. As the temperature gradually decreases to 25 °C, a pale white solid is obtained, which is dried at 70 °C for 5 h. The dried solids were ground, and BDD powder was obtained with a yield of 93.1%.

BDD: ^1^H NMR (400 MHz, DMSO-d6) δ 8.17 (dd, J = 16.5, 10.1 Hz, 4H), 8.04–7.71 (m, 4H), 7.51 (dd, J = 23.2, 15.1 Hz, 2H), 7.44–7.34 (m, 6H), 7.34–7.14 (m, 10H), 7.14–6.96 (m, 2H), 6.68–6.59 (m, 4H), 6.56–6.49 (m, 4H), 3.40 (t, J = 9.4 Hz, 2H) and 3.30 (s, 2H).

### 2.3. Preparation of BDD Modified EP

Table 1 lists the raw material ratios of EPs with different phosphorus contents. To begin, a certain quantity of BDD was introduced into the EP and underwent a reaction at a temperature of 150 °C for a duration of 30 min. This resulted in the formation of a light-yellow liquid, which was subsequently cooled down to 100 °C and combined with the pre-dissolved DDM. Subsequent to the mixing, the liquid was defoamed in a vacuum environment at 90 °C for 5 min. Later, the flame-retardant EP was placed into a glass mold and subjected to sequential curing at 80 °C for 2 h and 150 °C for 2 h.

### 2.4. Characterization

The FT-IR spectrum was measured by a Nicolet6700 spectrometer (Waltham, MA, USA) in the range of 4000–400 cm^−1^. Thirty-two scans were performed for a single spectrum with a spectral resolution of 4 cm^−1^. Meanwhile, ^1^H and ^31^P NMR spectra were obtained using DMSO-d6 as the solvent on a Bruker DRX 600 spectrometer (Billerica, MA, USA). X-ray photoelectron spectroscopy (XPS) was performed on an AXIS SUPRA spectrometer (Kyoto, Japan). These analytical techniques enabled us to determine the molecular structures and functional groups present in the sample.

DSC analysis was performed on a STA449F3. The nonisothermal curing kinetics of BDD-modified EP were studied at different heating rates (5, 10, 15 and 20 °C min^−1^) from 35 °C to 300 °C under nitrogen conditions.

Dynamic mechanical analysis (DMA) was performed on a Pyris Diamond instrument (Waltham, MA, USA). The sample size used in the experiment is 60 × 10 × 3 mm^3^. The instrument uses a bending mode and heats up from 30 °C to 225 °C at a rate of 10 °C min^−1^ at a frequency of 1 Hz.

A thermal gravimetric analyzer (TGA) experiment was conducted using a STA449F3 instrument (Selb, Germany). The experiment involved subjecting the sample to a temperature increase from 30 °C to 1000 °C at a constant rate of 10 °C per minute within an environment containing nitrogen. The purpose was to monitor the reduction in the weight of the sample.

The thermal decomposition test was carried out using NEZTSCH STA449F5 (Selb, Germany) in a nitrogen environment. The test temperature range was 25–800 °C, and the heating rate was 10 °C min^−1^. The synchronous FT-IR spectrum of pyrolysis products was acquired with the aid of a Bruker TGA-IR spectrometer.

The limiting oxygen index (LOI) of the sample with a size of 150 × 6.5 × 3 mm^3^ was tested on the JF-3 instrument (Beijing, China), according to ASTM D2863-19 According to ASTM D3801-20a, the vertical burning test was carried out on NK8017A (Dongguan, China) with a rectangular sample of 130 × 13 × 3 mm^3^. The cone calorimeter test, as specified by ISO 5660-1, was performed on a sample of 100 × 100 × 3 mm^3^ using the FTT machines. The external heat flux used was 35 kW/m^2^ [28,29,30].

The surface morphology of the samples was analyzed using a JSM-7500F scanning electron microscope (SEM) (Tokyo, Japan) with a 15 kV accelerated voltage after the flame-retardant test. EDS measured the carbon layer’s surface elements using an X-Max N80 spectrometer (Oxford, UK).

The tensile test was performed using an LE5105 machine (Shanghai, China) at a rate of 2 mm min^−1^ in accordance with ISO 527-1:2019, utilizing specimens measuring 150 × 10 × 4 mm³. Similarly, the flexural test was conducted using an LE3505 machine at a rate of 2 mm min^−1^ following ASTM D790-17 standards, with specimens measuring 80 × 10 × 4 mm³. The average of five samples was taken as the test result [31,32].

## 3. Results and Discussion

### 3.1. Structural Characterization

**Figure 2:**
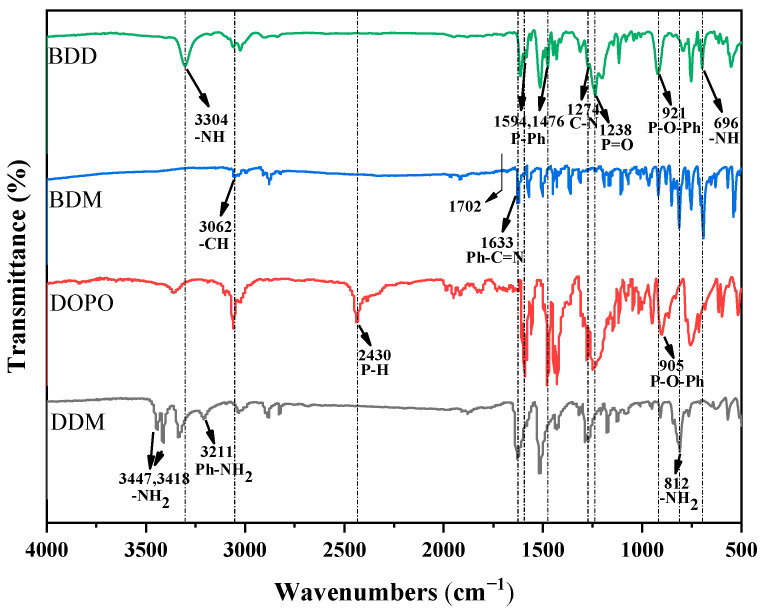
FTIR spectra of BDD, BDM, DDM and DOPO.

The FT-IR spectra of BDD, BDM, DOPO and DDM are shown in Figure 2. Firstly, DDM, benzaldehyde and the intermediate product BDM were analyzed. Benzaldehyde is one of the most commonly used aromatic aldehydes in industry. Its characteristic peaks are mainly located at 3065 cm^−1^, 2820 and 2738 cm^−1^ and 1702 cm^−1^, corresponding to the stretching vibration peaks of the -C-H bond on the benzene ring, the -C-H bond on the aldehyde group and -C=O, respectively. The group of DDM involved in the reaction was -NH_2_. It can be seen that the peaks of -NH_2_ at 3447, 3418 and 3211 cm^−1^ in BDM all disappeared, which indicates that -NH_2_ was successfully involved in the reaction. The peak at 3062 cm^−1^ is attributed to the hydrocarbon stretching vibration on the benzene ring of benzaldehyde. The stretching vibration peak of -C=O of benzaldehyde generally appears at approximately 1700 cm^−1^, but this peak is not observed in the spectrum of BDM, which indicates that -C=O on -CHO of benzaldehyde is also successfully involved in the reaction. In addition, the characteristic peak at 1633 cm^−1^ in BDM belongs to Ph-C=N, which also exists in DDM, so it is difficult to judge. However, combined with the disappearance of the characteristic peaks of the -C=O bond of benzaldehyde and the -NH_2_ bond of DDM, it can be concluded that BDM was successfully synthesized.

Subsequently, the FT-IR spectra of DDM, DOPO and BDD were analyzed. Compared with the spectrum of BDD, the bending vibration peak of -NH_2_ at 812 cm^−1^, the stretching vibration peak of -NH_2_ at 3447 cm^−1^ and 3418 cm^−1^ and the binding peak of the benzene ring and amino group at 3211 cm^−1^ in DDM were not observed in the BDD spectrum, indicating a successful reaction between DDM and benzaldehyde and the synthesization of the intermediate Schiff base [3]. Compared with the FT-IR spectrum of DOPO, the peaks of BDD at 1594 cm^−1^ and 1476 cm^−1^ corresponded to the stretching vibration of the P-Ph bond, and the characteristic absorption peaks of the P=O bond and P-O-Ph bond appeared at 1238 cm^−1^ and 921 cm^−1^, respectively [33]. The absorption peak of P-O-Ph in BDD is obtained by shifting the peak at 905 cm^−1^ in the DOPO spectrum to a high wavenumber [33]. The absorption peak of the P-H of the DOPO group near 2371 cm^−1^ did not appear in the BDD spectrum, and the peak of the C-N appeared at 1274 cm^−1^. In addition, the bending vibration peak of -NH at 696 cm^−1^ and the tensile vibration peak of-NH at 3304 cm^−1^ appear in the spectrum of BDD. This indicates that the P-H reacted with the Schiff base, confirming the synthesis of BDD.

**Figure 3:**
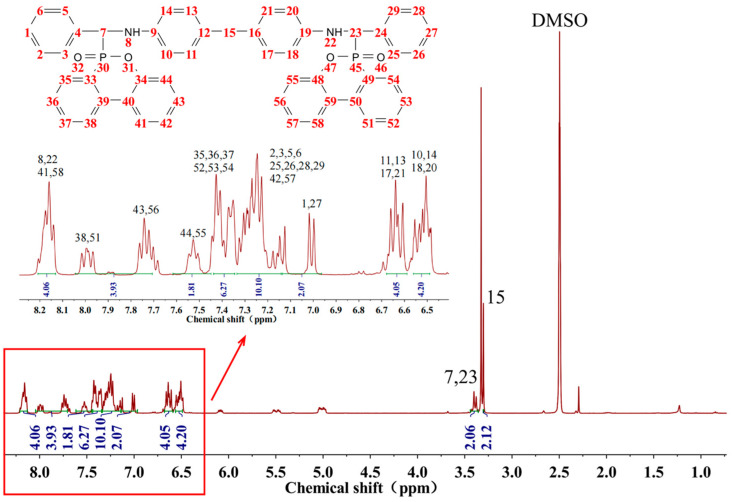
^1^H NMR spectra of BDD.

The ^1^H NMR spectrum of BDD (Figure 3) displays two protons located on the primary carbon at 3.30 ppm, two protons located on the tertiary carbon at 3.40 ppm, aromatic hydrogen located between 6.45 and 8.25 ppm and two imino groups at 8.18 ppm. The benzene ring and the phosphaphenanthrene group’s hydrogen are challenging to distinguish and can only be roughly identified. However, after integrating all the peak areas, the ratio of the three hydrogens (C-H:N-H:Ar-H) can be calculated to be 2:1:17, consistent with each element’s proportion in BDD. In addition, the impurity peak at approximately 2.2 ppm may be the characteristic peak of -CH_3_ in the residual toluene in benzaldehyde. This confirms that BDD has been successfully synthesized.

**Figure 4:**
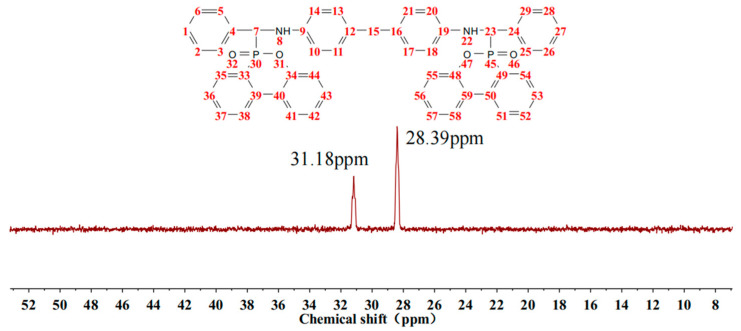
^31^P NMR spectra of BDD.

Figure 4 displays the ^31^P NMR spectra of BDD. The phosphorus peaks in DOPO molecules are near 31.18 ppm and 28.39 ppm, respectively [34]. The splitting of the phosphorus peak of BDD is due to the considerable steric hindrance of the DOPO group, and the BDD molecule forms different stereoisomers under the steric hindrance of the two DOPO groups [35,36].

**Figure 5:**
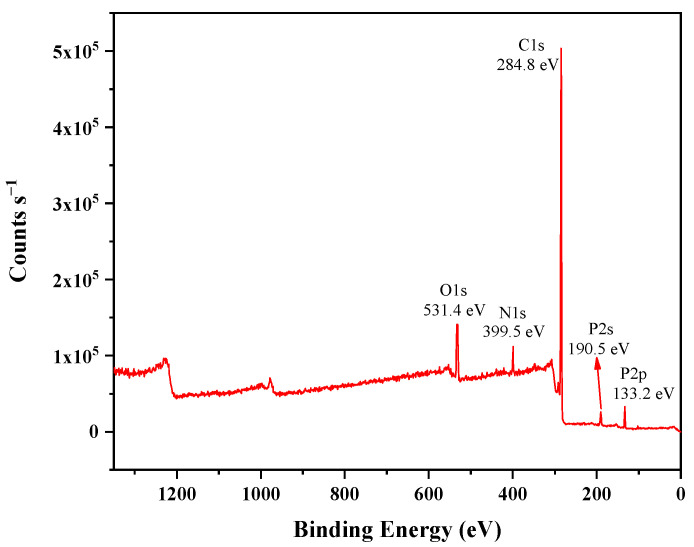
XPS spectrum of BDD.

The XPS spectrum of BDD is shown in Figure 5. According to the peak area, the ratio of C, O, N and P in the sample is 69.80:4.80:3.74:3.27. The corresponding theoretical ratio of C, O, N and P in the theoretical structure of BDD is 51:4:2:2. Although BDD may contain some impurities, such as incompletely reacted raw materials, which may cause the results to be different from the theoretical values, the two sets of data can be compared according to the cosine similarity. The calculation method is as follows:

(1) $$cos\theta =\frac{\left(x,y\right)}{\left|x\right|\left|y\right|}$$

Among them, *x* and *y* are vectors in the form of (*x*_1_, *x*_2_, *x*_3_ *· · · x_n_*), (*y*_1_, *y*_2_, *y*_3_ *· · · y_n_*), *x_i_* and *y_i_* are the proportions of each element calculated according to the BDD theoretical structure and XPS data, respectively. The value range of cosine similarity is [−1,1]. According to Formula 2.1, the similarity between the theoretical structure of BDD and the proportion of elements in XPS data is 0.9998. The closer the result is to 1, the higher the similarity is and the smaller the deviation is. Therefore, the XPS data show that the proportion of elements in BDD is highly consistent with its theoretical structure. This is an effective supplement to the results of FT-IR, ^1^H NMR and ^31^P NMR.

### 3.2. Analysis of Curing Behavior

**Figure 6:**
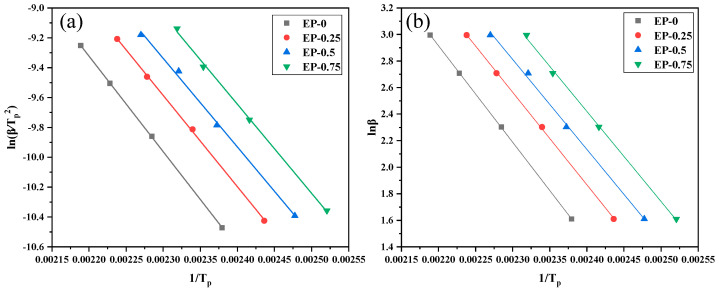
Two linear relationships obtained by Kissinger (**a**) and Ozawa (**b**) methods.

The nonisothermal curing kinetics of the EP/BDD/DDM system were studied by DSC. The activation energy of the curing reaction can be calculated using the formula of Kissinger and Ozawa [37,38]. Kissinger’s formula is as follows:

(2) $$-\ln\left(\beta /T_{p}^{2}\right)=E_{a}/{RT}_{p}-\ln\left(AR/E_{a}\right)$$

where $E_{a}$ is the apparent activation energy, R is the ideal gas constant, β is the heating rate, A is the pre-exponential factor and $T_{p}$ is the exothermic peak temperature. The peak exothermic temperatures of epoxy-cured products with different phosphorus contents measured in the test are summarized in Table 2. The linear relationship between $\ln\left(\beta /T_{p}^{2}\right)$ and $1/T_{p}$ calculated according to Kissinger’s method is shown in Figure 6a. The Ozawa method is shown in Equation (3).

(3) $$\ln\beta =-1.052\left(E_{a}/{RT}_{p}\right)+\ln\left(AE_{a}/R\right)-\ln F\left(\alpha \right)-5.331$$

The linear relationship between ln⁡β and $1/T_{p}$ obtained from the above equation is shown in Figure 6b. As shown in Table 3, both methods can be used to calculate the apparent activation energy of the EP system by the slope of the corresponding line. It can be seen that the addition of BDD effectively reduces the activation energy of the EP system and improves the curing activity, which is because the imino group on BDD contains reactive active hydrogen. The hydrogen in the imino group can also form hydrogen bonds with the oxygen atoms in the epoxy group, which is beneficial to the ring-opening reaction of the epoxy group.

### 3.3. Thermal Properties

**Figure 7:**
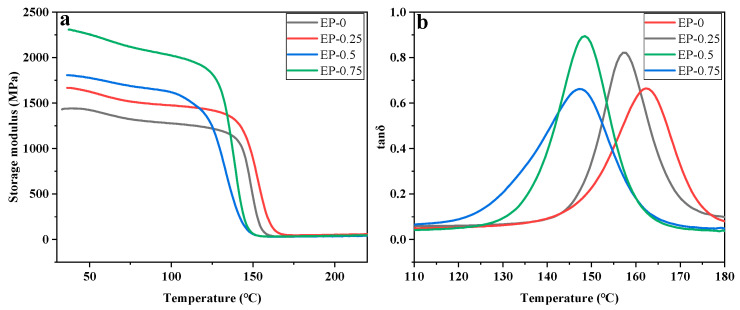
Storage modulus (**a**) and tanδ (**b**) of cured epoxy resins with different phosphorus contents.

Figure 7 shows the storage modulus (E′) and loss tangent (tanδ) of EPs. Correspondingly, the storage modulus (at 35 °C), T_g_ and crosslinking density (ν_e_) of each resin are presented in Table 4. The DMA results demonstrated that the incorporation of BDD led to an augmentation in the E′ of EPs. For the phosphorus content of 0.75%, the storage modulus of EP-0.75 is 2296.9 MPa, 59.5% higher than EP-0. The stiffness of the EP network is increased by the substantial rigidity of the aromatic rings of BDD, as indicated by the storage modulus. The peak value of different tanδ curves in Figure 7 represents the T_g_ of EP with additional phosphorus content. As the phosphorus level increases, the T_g_ of EP shows a downward trend, while the storage modulus is the opposite. In general, T_g_ depends on the stiffness of the polymer structure and its crosslinking density (ν_e_) [39]. Based on the principles of rubber elasticity theory, the ν_e_ can be determined using the following mathematical formula [40]:

*ν_e_* = *E*′/3*RT* (4)

where *E′* is the storage modulus at T_g_ +45 °C, *R* is the ideal gas constant, and *T* is the thermodynamic temperature at T_g_ + 45 °C. It is evident from Table 4 that a rise in BDD content leads to a decrease in ν_e_ of EP. Although the rigid group in BDD will reduce the activity of the EP segment and lead to an increase in T_g_, the crosslinking density of the EP may be reduced under the influence of steric hindrance. Additionally, BDD, which has only two reactive hydrogens, reduces the bonding sites of EP, resulting in a further reduction in its crosslinking density. The T_g_ of EP falls when BDD is added, owing to the combined influence of these factors.

**Figure 8:**
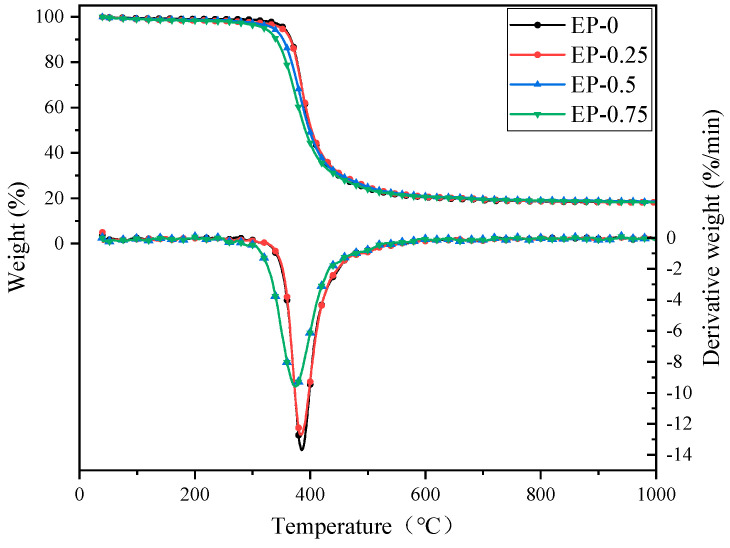
TGA and DTG curves of cured epoxy resins at different phosphorus contents.

The TGA and DTG curves of EP are shown in Figure 8, with the data of T_5%_ (5% weight loss temperature), T_max_ (maximum weight loss rate temperature), R_max_ (maximum weight loss rate) and R_700_ (residual mass at 700 °C) collected in Table 5. The T_5%_ of EP exhibits a steady decline with increasing phosphorus levels. This is because the flame retardant BDD contains an unstable O=P-O bond [41], causing it to decompose at a lower temperature and generate substances such as phosphoric and polyphosphoric acids. The DTG curves in Figure 8 exhibit a single sharp weight loss peak for each resin in a nitrogen atmosphere, with R_max_ decreasing as the phosphorus level increases. The decrease in R_max_ is due to the instability of BDD macromolecules, which will undergo pyrolysis at a lower temperature. The pyrolysis products of BDD are evenly distributed on the surface of EP, which promotes the formation of a protective carbon layer and prevents its further decomposition. Therefore, as the proportion of BDD in the EP system increases, the R_700_ of the cured EP gradually increases, exhibiting gradually enhanced thermal stability of the EP.

### 3.4. Flame-Retardant Property

**Figure 9:**
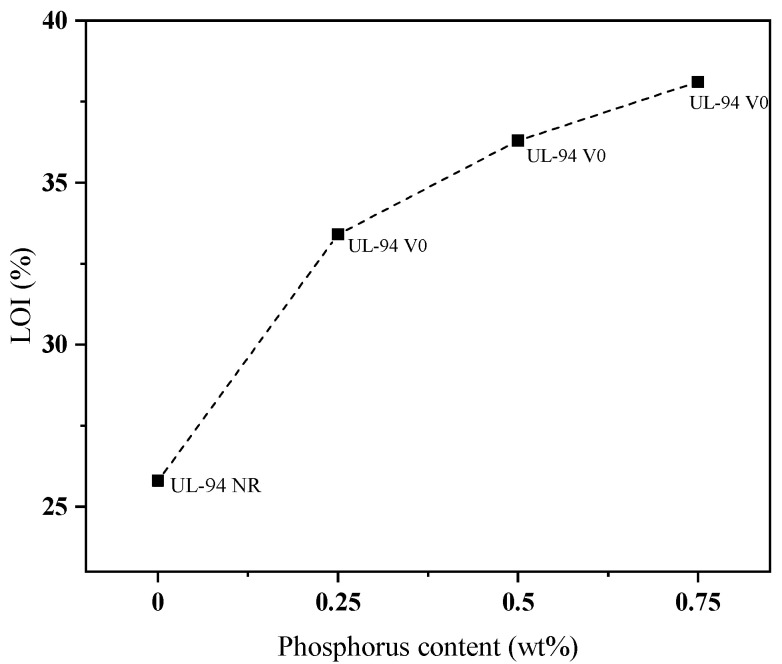
LOI and UL-94 rating of the cured epoxy resins with different phosphorus contents.

Figure 9 shows the vertical burning rating and LOI value of EP with different BDD additions, which are elaborated in Table 6. The flame-retardant qualities of BDD-modified EP are very commendable. An EP-0.25 material can earn a V-0 rating in the UL-94 classification when its phosphorus concentration is limited to 0.25 wt%. The LOI value of EP-0 without BDD was 25.8%. Nevertheless, it exhibits a significant increase when BDD is introduced, ultimately peaking at 38.1% when the percentage of phosphorus reaches 0.75 wt% [42].

Phosphorus is the essential ingredient that imparts flame retardancy to EP in BDD. During combustion, PO· released by BDD decomposition can effectively capture the surrounding active free radicals, thereby reducing the energy of the flame. Furthermore, the presence of phosphate and polyphosphoric acid in the condensed phase facilitates the creation of a compact carbon layer, effectively shielding the polymer from both heat and air [43,44]. Moreover, the nitrogen-containing groups produce nonflammable gases when burned, which have a flame-retardant action in the gas phase [45]. Therefore, incorporating BDD into the EP results in a remarkable enhancement of flame retardancy. Xu et al. [3] synthesized D-bp with a molecular structure similar to BDD to modify EP. When the phosphorus content of the modified resin is 0.25 wt %, the LOI value of EP is 30.5%, which can pass UL-94 V-1 certification. By comparing the LOI value and UL 94 test results at the same phosphorus content, it can be determined that the flame-retardant efficiency of BDD is higher than that of D-bp. The only difference between the two flame retardants is that D-bp has two extra hydroxyl groups. In the initial stage of the thermal decomposition of EP, the nonchain-breaking reactions of dehydrogenation and dehydration occur, forming an unstable double bond related to the secondary alcohol group in the EP [46]. However, the hydroxyl groups in D-bp are not entirely reacted, and they promote the generation of methane, carbon dioxide, formaldehyde and hydrogen during combustion, which are flammable gases except for carbon dioxide. Thus, the EP cured with BDD, which does not contain a hydroxyl group, is more effective in terms of flam-retardant properties than D-bp. It can be inferred that the elimination of certain unstable groups from the current flame retardants is a feasible strategy to enhance efficiency.

**Figure 10:**
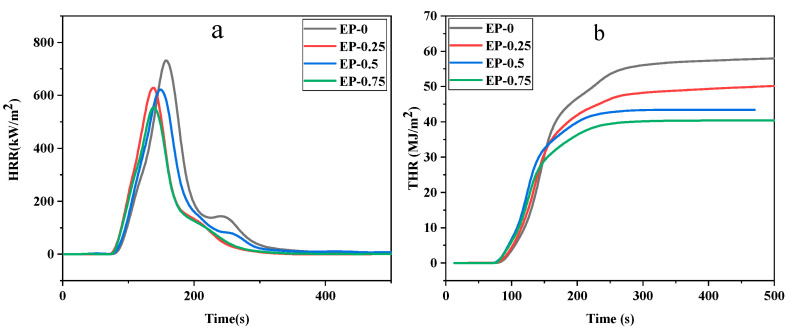
Heat release rates (**a**) and total heat release (**b**) of the cured epoxy resins with different phosphorus contents.

The cone calorimeter test is employed to further assess the combustibility of EP. The requested data include heat release rate (HRR), peak heat release rate (P-HRR), total heat release (THR), effective heat of combustion (EHC) and time to ignition (TTI). Figure 10 displays the relationship between the HRR and the THR over time for various phosphorus concentrations in the EP. The P-HRR and EHC data in Table 7 showed that with the addition of BDD, the P-HRR and THR of EP decreased significantly with the increase in phosphorus content, and EHC also decreased gradually. BDD offers a prosperous enhancement to the flame retardancy of EP. Table 7 demonstrates that the TTI of EP-0 was 55 s, and that of EP-0.75 declined to 44 s with a 20% reduction. This is because the phosphorus-containing groups begin to pyrolyze at lower temperatures and subsequently catalyze the creation of a durable carbon layer on the surface of the polymer [39]. As for the residue, EP-0 was almost completely burned, with a mass loss of 94.37% or so. The addition of BDD resulted in a notable rise in the char amount of the resins, which confirms the mechanism by which BDD facilitates the creation of a carbon layer so as to isolate heat and limit the emission of flammable gases [47].

It is crucial to highlight that the residue of EP-0.25 shows a substantial increase when compared to EP-0. The quantity of residual carbon remains nearly constant as the phosphorus content steadily rises. The phosphorus content does not appear to dramatically affect the char yield. This is because more EP has been pyrolyzed, forming a dense carbon layer. The experiments reveal that a small amount of BDD improves the flame retardancy of EP effectively. Nevertheless, increasing the percentage of BDD does not result in an enormous leap in the flame-retardant properties of EP. Therefore, the inclusion of a minimal quantity of BDD can effectively enhance the flame-retardant properties of EP without compromising its mechanical capability.

### 3.5. Morphologies and Components of Char Residue

**Figure 11:**
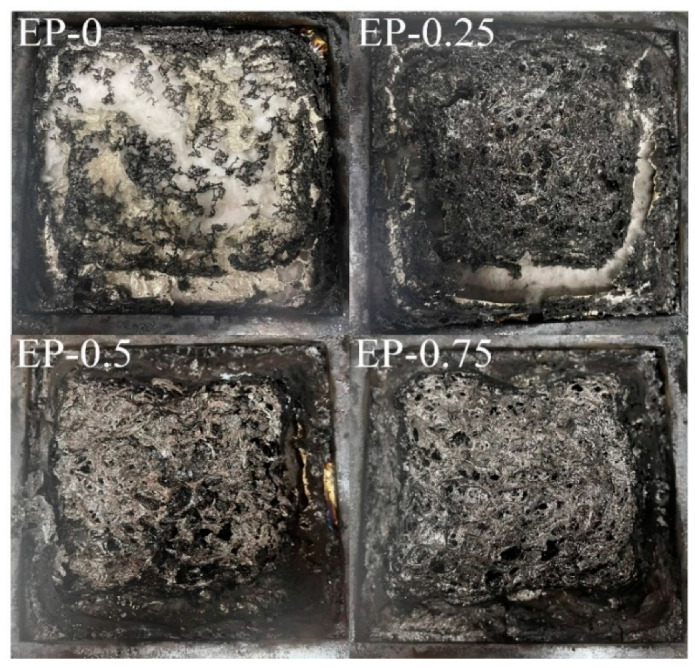
Digital photos of the residues after the cone calorimeter test.

The photos of the residual of the EP sample following the cone calorimeter test are displayed in Figure 11. Only scattered residual carbon can be observed in EP-0. In contrast, the EP incorporated with BDD retained its cubic shape after combustion, demonstrating the role of BDD in enhancing and accelerating char residue formation.

**Figure 12:**
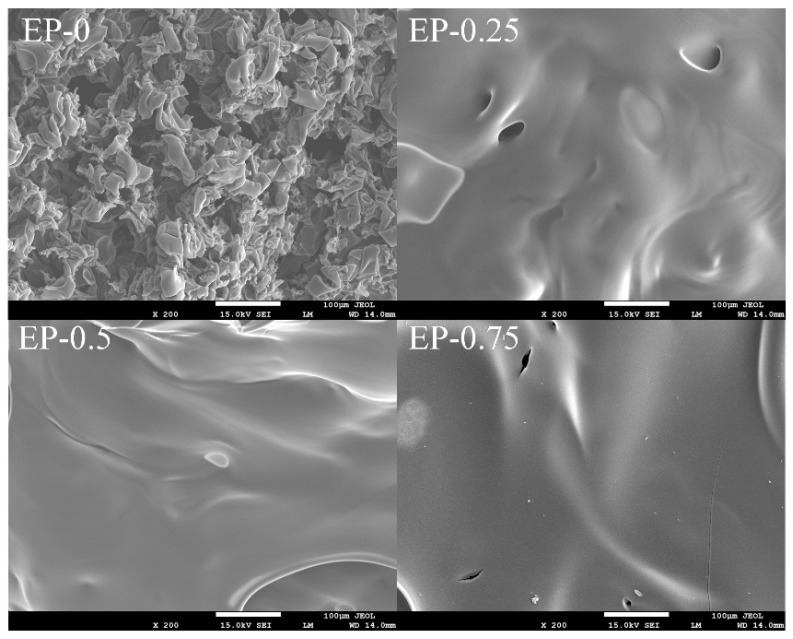
SEM photographs of the cured epoxy resins with different phosphorus contents.

**Figure 13:**
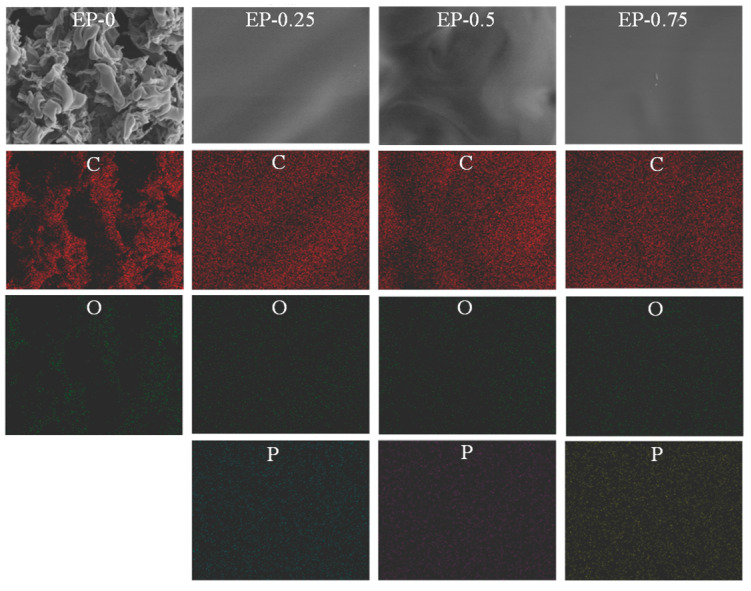
SEM micrographs and merged EDS elemental mapping images of the cured epoxy resins with different phosphorus contents.

Figure 12 displays the microscopic structure of residual carbon as observed through the cone calorimeter. The results indicate that EP-0 is severely damaged, with a charred surface full of holes and cracks that help transfer oxygen and heat. Conversely, the carbon residue surface of EP containing BDD is smooth and dense, with only a tiny number of micropores, which is consistent with the observations shown in Figure 11. The EP containing BDD forms a dense carbon layer that successfully blocks the heat transfer during combustion [21], thereby showing excellent flame retardancy. The elemental distribution of chars was analyzed using EDS element mapping, as shown in Figure 13. The element mapping corresponding to the external residual carbon shows that C, O and P are evenly distributed, which may be related to the carbonization catalysis of phosphorus and the quenching effect of PO free radicals, indicating that the phosphorus-containing macromolecules are pyrolyzed into phosphorus-containing derivatives such as phosphoric acid during combustion and uniformly attached to the surface of the substrate to catalyze dehydration and dehydrogenation, thus forming a dense carbon layer [48,49]. This discovery offers more substantiation for the effectiveness of integrating phosphorus into the EP as a flame retardant.

### 3.6. TG-IR Analysis

**Figure 14:**
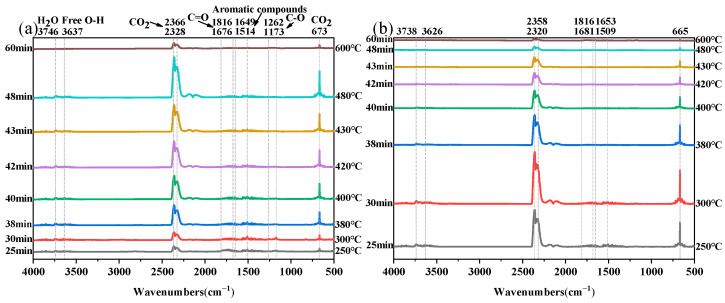
FTIR spectra of the pyrolysis products for EP-0 (**a**) and EP-0.25 (**b**) at different times.

**Figure 15:**
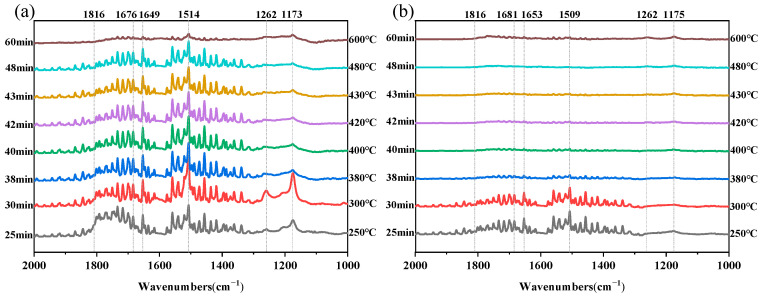
Partial FTIR spectra of the pyrolysis products of EP-0 (**a**) and EP-0.25 (**b**) at different times.

The volatile components of EPs (EP-0 and EP-0.25) were continually monitored using TGA-FTIR. As depicted in Figure 14a and Figure 15a, the primary gaseous products of pure EP volatiles include H_2_O (3746 cm^−1^), free O-H (3637 cm^−1^), CO_2_ (2366, 2328 and 673 cm^−1^), carbonyl C=O (1816−1676 cm^−1^), aromatic compounds (1649 and 1514 cm^−1^) and ester or ether components (1262 and 1173 cm^−1^) [50,51]. Aromatic amine, phenolic and carbonyl-containing substances are the primary components of these structures [52], in which carbonyl-containing substances are highly flammable volatile fuels.

As observed in Figure 15a,b, the main absorption peaks of EP-0.25 are essentially the same as those of EP-0, except for the different peak intensities in the corresponding regions. The area of the peak can represent the amount of the corresponding volatiles. It can be seen that a significant amount of CO_2_ was generated during the initial phase of EP-0.25 combustion, with the maximum reached at 30 min or so, when CO_2_ had just begun to generate for EP-0. This is strongly correlated with the TGA results above. The phosphorus-containing functional group is unstable, and it rapidly decomposes into free radicals during the initial moments of burning and catalyzes the pyrolysis of EP to form a dense protective carbon layer, which inhibits further combustion. Due to the lack of an effective protective carbon layer formed at the initial reaction stage, the pure EP will quickly burn out as the reaction intensifies. In addition, the absorption peaks of EP-0.25 at 1816–1676 cm^−1^, 1649 cm^−1^, 1514 cm^−1^, 1262 cm^−1^ and 1173 cm^−1^ are much lower than those of EP-0. Carbonyl C=O (1816−1676 cm^−1^), aromatic compounds (1649 and 1514 cm^−1^) and ether components (1173 cm^−1^) are considered to be combustible gases released during the combustion of epoxy. This phenomenon can be explained by the fact that the PO· free radicals, which are produced when phosphorus-containing flame retardants undergo pyrolysis, are able to capture O· free radicals. This reduces the attack on the carbon chain and leads to a decrease in the oxygen content of the air, which further inhibits the combustion of the EP and reduces the combustible gases from the pyrolysis of the carbon chain. The dense carbon layer formed due to the incorporation of the flame retardant also inhibits further cracking of the EPs. These factors play together and lead to a significant reduction in the emission of combustible gases, which also offers flame-retarding effects in the gas phase.

### 3.7. Mechanical Properties

**Figure 16:**
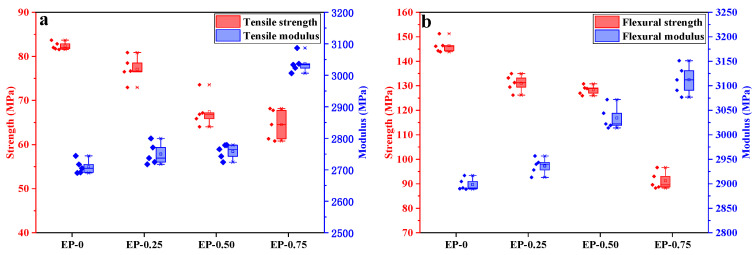
The tensile (**a**) and flexural (**b**) properties of the cured epoxy resins with different phosphorus contents.

Figure 16 illustrates a comparison of the mechanical characteristics of EPs with varying phosphorus amounts. The mean and standard deviation of the mechanical properties of each sample are shown in Table 8. The mechanical properties of EPs show a consistent pattern. As the phosphorus content increases, the tensile and flexural strengths steadily fall, but the tensile and flexural moduli increase. For D-bp with a similar structure to BDD, when the D-bp modified EP reached UL 94 V-0 level, the tensile and flexural strengths decreased by 13.1% and 10.3% compared to EP-0, respectively. In this paper, as far as EP-0.25 is concerned, the V-0 rating in the UL-94 test is achieved with the average tensile and flexural strengths of 77.1 MPa and 131.02 MPa, respectively, only 6.4% and 10.5% lower than those of the EP-0. However, the tensile and flexural strengths of EPs decrease significantly for higher phosphorus contents of 0.5 wt% and 0.75 wt%. This is attributed to the rigid structures, such as benzene and heterophenanthrene rings, introduced into the epoxy-cured network by BDD [53]. On one hand, the molecular structures of BDD occupy the crosslinking sites of the EP network. This reduces the crosslinking density of EP and leads to a decline in both its tensile strength and modulus. On the other hand, these macromolecular structures have considerable steric hindrance, resulting in a decrease in the flexibility of molecular chains and an increase in moduli. The weak bonds in the phosphorus-containing groups such as O=P-O and P-C on BDD also cause an elevation in stiffness and a reduction in strength, making the cured epoxy products more brittle and less tough [41]. For the combined consideration of fire retardancy and mechanical behavior, it is suggested that the optimal amount of phosphorus content is 0.25 wt% for the flame-retardant EP.

## 4. Conclusions

To summarize, a reactive flame retardant BDD based on DOPO was synthesized by a two-step technique, and its structure was verified. Different quantities of BDD were added to manufacture a variety of flame-retardant EPs. As a reactive flame retardant, the addition of BDD reduces the apparent activation energy of the EP system. Compared with the EP-0, the remarkable flame retardancy of the EP-0.25 is achieved with the V-0 rating of UL-94 and the LOI value of 33.4%. The P-HRR and THR are reduced by 9.1% and 14.5%, respectively. Although the T_g_ and initial degradation temperature of EP-0.25 are lower than those of EP-0, the storage modulus and residual carbon content increase significantly at high temperatures. Furthermore, higher tensile and flexural moduli are exhibited by EP-0.25, with similar strengths maintained compared to EP-0. The flame-retardant mechanism of BDD primarily involves such effects as the quenching of phosphorus-containing free radicals, the dilution of noncombustible gases released during combustion and the barrier of an expanded dense carbon layer. Therefore, compared with DOPO-based flame retardants reported so far, a small amount of BDD can effectively endow EP with flame retardancy. However, a large number of rigid groups in BDD still affect the glass transition temperature and mechanical properties of EP. It is anticipated that BDD will be a highly effective flame retardant without halogen for EPs used in commercial applications.

## Figures and Tables

**Table 1:** Recipes for the flame-retarded epoxy resins.

Sample	DGEBA (g)	DDM (g)	BDD (g)	P (wt%)	N (wt%)
EP-0	100	25	0	0	2.83
EP-0.25	100	24.73	4.19	0.25	2.71
EP-0.5	100	24.19	8.64	0.50	2.58
EP-0.75	100	23.60	13.35	0.75	2.44

**Table 2:** The exothermic peak temperature of cured epoxy resin at different heating rates.

Heating Rate (°C/min)	EP-0 (°C)	EP-0.25 (°C)	EP-0.5 (°C)	EP-0.75 (°C)
5	147.13	137.32	130.50	123.57
10	164.52	154.24	148.33	140.71
15	175.65	165.79	157.71	151.63
20	183.71	173.68	167.39	158.13

**Table 3:** The apparent activation energy of cured epoxy resins at different phosphorus contents.

Sample	E_a1_ (kJ/mol)	R^2^	E_a2_ (kJ/mol)	R^2^
EP-0	53.2	0.999	57.5	0.999
EP-0.25	50.8	0.999	55.1	0.999
EP-0.5	49.9	0.997	53.5	0.998
EP-0.75	49.4	0.999	53.0	0.999

E_a1_: calculated by Kissinger’s method. E_a2_: calculated by Ozawa’s method.

**Table 4:** Storage modulus at 40 °C, glass transition temperature, and crosslink density of the cured epoxy resins with different phosphorus contents.

Sample	E′ (35 °C) (MPa)	T_g_ (°C)	ν_e_ (mol/m^3^)
EP-0	1439.7	162.4	4488
EP-0.25	1661.2	157.6	4390
EP-0.5	1800.5	148.5	4355
EP-0.75	2296.9	147.1	4274

**Table 5:** TGA data of cured epoxy resins at different phosphorus contents.

Sample	T_d5%_ (°C)	T_max_ (°C)	R_max_ (%/min)	R_700_ (%)
EP-0	355	385	−13.68	19.22
EP-0.25	350	384	−12.69	19.54
EP-0.5	336	382	−10.31	19.86
EP-0.75	320	375	−9.58	20.03

**Table 6:** Fire retardancy of the cured epoxy resins with different phosphorus contents.

Sample	P (wt%)	LOI Values (%)	UL-94 Rating	Drip
EP-0	0	25.8	NR	Yes
EP-0.25	0.25	33.4	V0	No
EP-0.5	0.5	36.3	V0	No
EP-0.75	0.75	38.1	V0	No

**Table 7:** Cone calorimeter test results of the cured epoxy resins with different phosphorus contents.

Sample	TTI (s)	P-HRR (kW/m^2^)	THR (MJ/m^2^)	Av-EHC (MJ/kg)	Residue (wt%)
EP-0	55	836.31	57.48	15.71	5.63
EP-0.25	49	760.53	49.13	14.62	13.49
EP-0.5	46	734.33	43.38	13.43	13.61
EP-0.75	44	675.24	40.34	12.45	14.73

**Table 8:** Mean and standard deviation of mechanical test data of the cured epoxy resins with different phosphorus contents.

Sample	Tensile Strength		Tensile Modulus		Flexural Strength		Flexural Modulus	
	Avg (MPa)	σ	Avg (MPa)	σ	Avg (MPa)	σ	Avg (MPa)	σ
EP-0	82.37	0.88	2709.45	22.38	146.39	2.95	2898.37	12.11
EP-0.25	77.10	2.91	2749.87	34.60	131.02	3.38	2936.29	16.55
EP-0.5	67.50	3.60	2757.77	23.71	128.33	1.91	3034.21	23.98
EP-0.75	64.52	3.46	3037.32	29.93	91.23	3.84	3112.28	29.95

## Data Availability

Data are contained within the article.
